# Prevalence of diabetes in the USA from the perspective of demographic characteristics, physical indicators and living habits based on NHANES 2009-2018

**DOI:** 10.3389/fendo.2023.1088882

**Published:** 2023-03-07

**Authors:** Ling Fang, Huafang Sheng, Yingying Tan, Qi Zhang

**Affiliations:** ^1^ Shaanxi Key Laboratory of Chinese Medicine Encephalopathy, Shaanxi University of Chinese Medicine, Xianyang, China; ^2^ Department of Laboratory Medicine, Zhujiang Hospital of Southern Medical University, Guangzhou, China

**Keywords:** NHANES, diabetes, leg length, BMI, total cholesterol, living habits

## Abstract

**Objective:**

To determine differences in DM in the U.S. population according to demographic characteristics, physical indicators and living habits.

**Methods:**

23 546 participants in the 2009 to 2018 National Health and Nutrition Examination Survey (NHANES) who were 20 year of age or older and not pregnant. All analyses used weighted samples and considered the stratification and clustering of the design. Specific indicators include length of leg (cm), BMI (kg/cm^2^), TCHOL (mg/dL), fasting plasma glucose (mg/dL) and comparison of means and the proportion of participants with DM.

**Results:**

The prevalence of DM in the USA has been rising modestly in the past decade, and were consistent and robust for the observed differences in age, sex, and ethnicity. Compared with white participants, black participants and Mexican-American were both more likely (P<0.001) to have diabetes: 14.6% (CI, 13.6% to 15.6%) among black participants, 10.6% (CI, 9.9% to 11.3%) among white participants, and 13.5% (CI, 11.9% to 15.2%) among Mexican-American participants. The prevalence of diabetes is increasing with age, males peaked around the 60s, and women around the 70s. The overall mean leg length and TCHOL was lower in diabetics than in non-diabetics (1.07 cm, 18.67 mg/dL, respectively), while mean BMI were higher in diabetics than in non-diabetics (4.27 kg/cm2). DM had the greatest effect on decline of TCHOL in white participants (23.6 mg/dL), less of an effect in black participants (9.67 mg/dL), and the least effect in Mexican-American participants (8.25 mg/dL). Notably, smoking had great effect on percent increment of DM in whites (0.2%), and have little effect on black and Mexican-Americans.

**Conclusions:**

DM is more common in the general population than might be clinically recognized, and the prevalence of DM was associated to varying degrees with many indicators of demographic characteristics, physical indicators, and living habits. These indicators should be linked with medical resource allocation and scientific treatment methods to comprehensively implement the treatment of DM.

## Introduction

Diabetes mellitus (DM) is a chronic metabolic disease with a series of metabolic disorders (glucose, protein, fat, water, electrolyte, etc.) and chronic deficiency, or dysfunction of blood glucose level or dysfunction caused by a variety of pathogenic factors, which has been an important public health problem in the whole world (1). In the past 4 decades, doctors have mainly observed the changes of blood glucose through fasting plasma glucose (FPG), glycated hemoglobin (HbA1c), 2h postprandial blood glucose (2hPG), to provide a basis for the diagnosis of diabetes (2, 3). Diabetes symptoms can be observed in individuals of different gender, race and age. South Asia ethnicity with diabetes have higher mortality compared with white Europeans, South Asian women were in particular affected (4, 5).

Obesity poses a huge risk on diabetes independently in the United States (6). Previous studies were mainly limited to differences in the type of diabetes by ethnic groups, and comparative analysis in aspects of fatality. The more representative judgment indicators of diabetes include leg length, BMI (Body Mass Index), and total cholesterol (total cholesterol) (7). Studies on the differences of these indicators under different races, age, gender and other factors are still lacking and not systematic enough (6, 8). Studies have shown that leg length has been associated with diabetes prevalence (7), which urgently needs to be tested by quantitative analysis of large samples. We are unaware of a systematic analysis regarding the population-based prevalence of DM.

The National Diabetes Data Group (NDDG) established a classification based mainly on diabetes treatment requirements, which was widely recognized and still used today. This classification included insulin dependent diabetes mellitus (IDDM), non-insulin dependent diabetes mellitus (NIDDM), gestational diabetes and diabetes secondary to other diseases like pancreatic cancer and other endocrine diseases (9). In our study, diabetes in pregnant women was excluded from the study to ensure general applicability (7). We used data from the 2009 to 2018 National Health and Nutritional Examination Survey (NHANES) to describe the prevalence of DM in the USA from the perspective of demographic characteristics, physical indicators and living habits, including age-, sex-, ethnicity-, smoking-, alcohol drinking-related differences in leg length, BMI and TCHOL in the United States, and focusing on the fasting plasma glucose (FPG) in black persons.

## Methods

**Figure 1:**
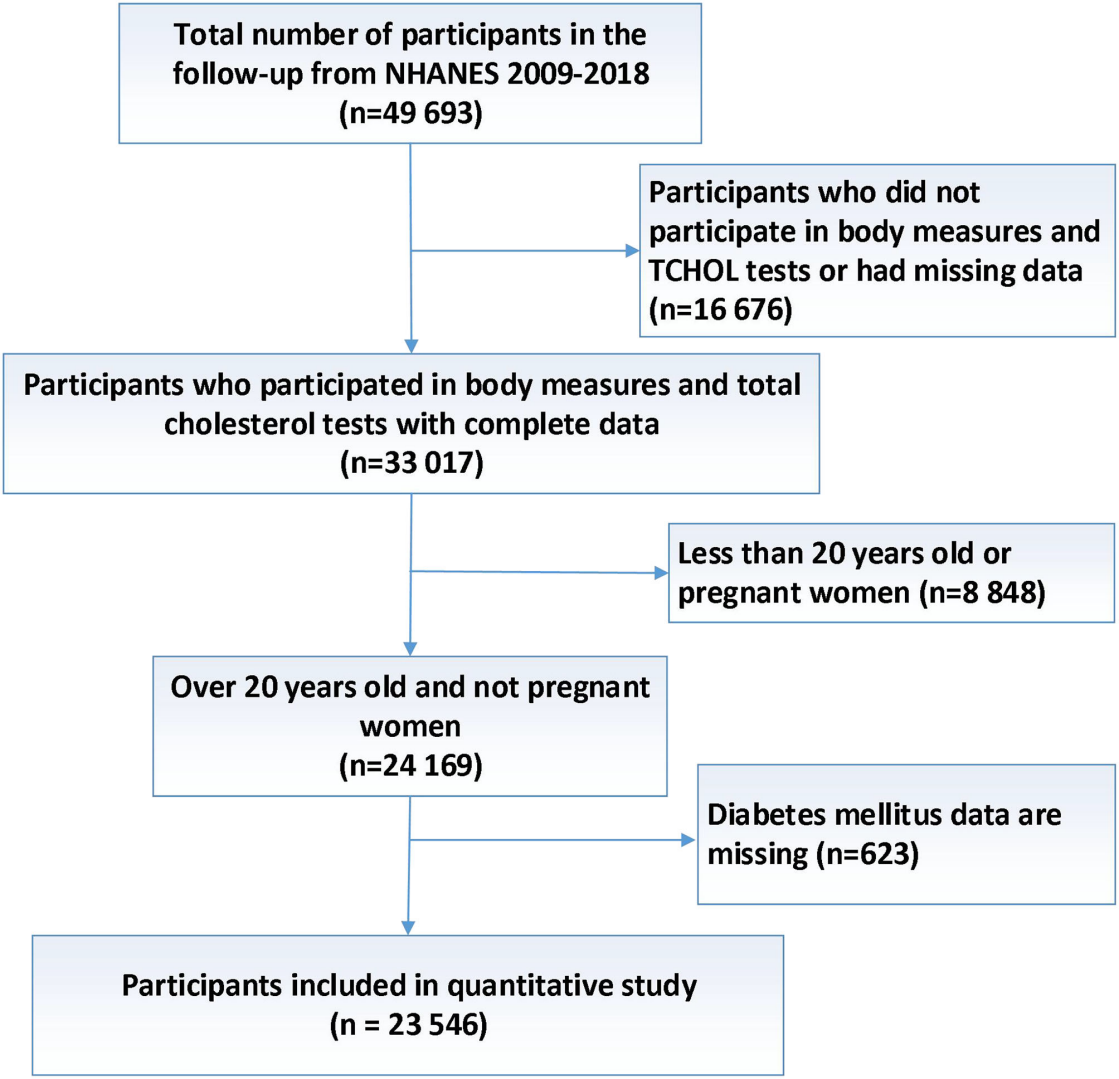
Flow diagram of participants inclusion for study.

Details of NHANES have been described elsewhere (10). In short, NHANES uses a complex, multistage, probabilistic sampling method to collect nationally representative health related data on the U.S. population, regular before 1999 and continued thereafter. Data were obtained through face-to-face interview, mobile physical examination and laboratory test. Since 2007, some changes have been made to the over-sampled domain. The main change was an oversampling of the entire Hispanic population, not just Mexican Americans (MA). Similar to those aged 60 and over in the previous cycle, blacks and low-income people were over-sampled to ensure accurate estimates for these groups (11). Of the 49 693 persons 1 year of age or older who were asked to come to the mobile examination centers, 16 676 did not participate in body measures and TCHOL tests or had missing data. 8848 persons were excluded because our study focused on people over 20 years old and no pregnant women, and 623 were excluded for DM data missing. Finally, 23 546 participants were included with complete data for quantitative study (**Figure 1**).

For the data sources and the selection criteria, three aspects should be explained. Firstly, leg length and BMI data from body measures of NHANES were collected in the Mobile Examination Center (MEC), by trained health technicians, in order to examine the associations between body weight and the health and nutritional status of the U.S. population (12). The laboratory method used to measure the value of TCHOL is an enzymatic assay, in which esterified cholesterol is converted to cholesterol by cholesterol esterase (13). Secondly, diabetes as diagnosed according to the standards of the American Diabetes Association (14) and participants’ self-reported questionnaires. Each of the following conditions was diagnosed as diabetes: fasting plasma glucose>=7 mmol/L (equal to 125 mg/dL), self-reported physician diagnosis of diabetes, or current use of diabetes medication to lower blood glucose level. Thirdly, current smokers were defined as participants who were age 20 years or older and reported smoking occasionally or daily during the past 7 days, or over 100 cigarettes (15). For alcohol drinking group, current heavy alcohol use (≥3 drinks per day for females, ≥4 drinks per day for males, or binge drinking [≥4 drinks on same occasion for females, ≥5 drinks on same occasion for males] on 5 or more days per month); current moderate alcohol use (≥2 drinks per day for females, ≥3 drinks per day for males, or binge drinking ≥2 days per month); all other cases are mild (16).

All analyses used weighted samples and considered the stratification and clustering of the design to derive estimates that were applicable to the U.S. population (12, 13). To provide estimates for the entire 10 years, a 10-year, weight-variable sample was created by taking one fifth for the 2-year weight for each person who was sampled in 2009 to 2018. All analyses were conducted in RStudio, version 2022.07.0 for Windows (RStudio, PBC) and R, version 4.2.1 (The R Foundation for Statistical Computing). Non-adjusted frequency distribution of body measures and TCHOL and non-adjusted prevalence of diabetes regarding age groups, sex, and ethnicity were obtained. These age groups were commonly used in highly stratified NHANES analyses (12, 13). We obtained means and comparisons of age-adjusted and sex-adjusted body measures by using linear regression, in which age is modeled as a continuous variable. Adjusted means in leg length and BMI in smokers and nonsmokers were compared. Logistic regression adjusting for age group and sex was then used to generate predictive marginals for having diabetes. More complex models, which involved interaction between ethnicity and sex as well as between ethnicity and age (as a continuous variable), were also tested and different categorizations of age were considered. These models did not substantially add to the explanatory power of the model.

## Results

**Figure 2:**
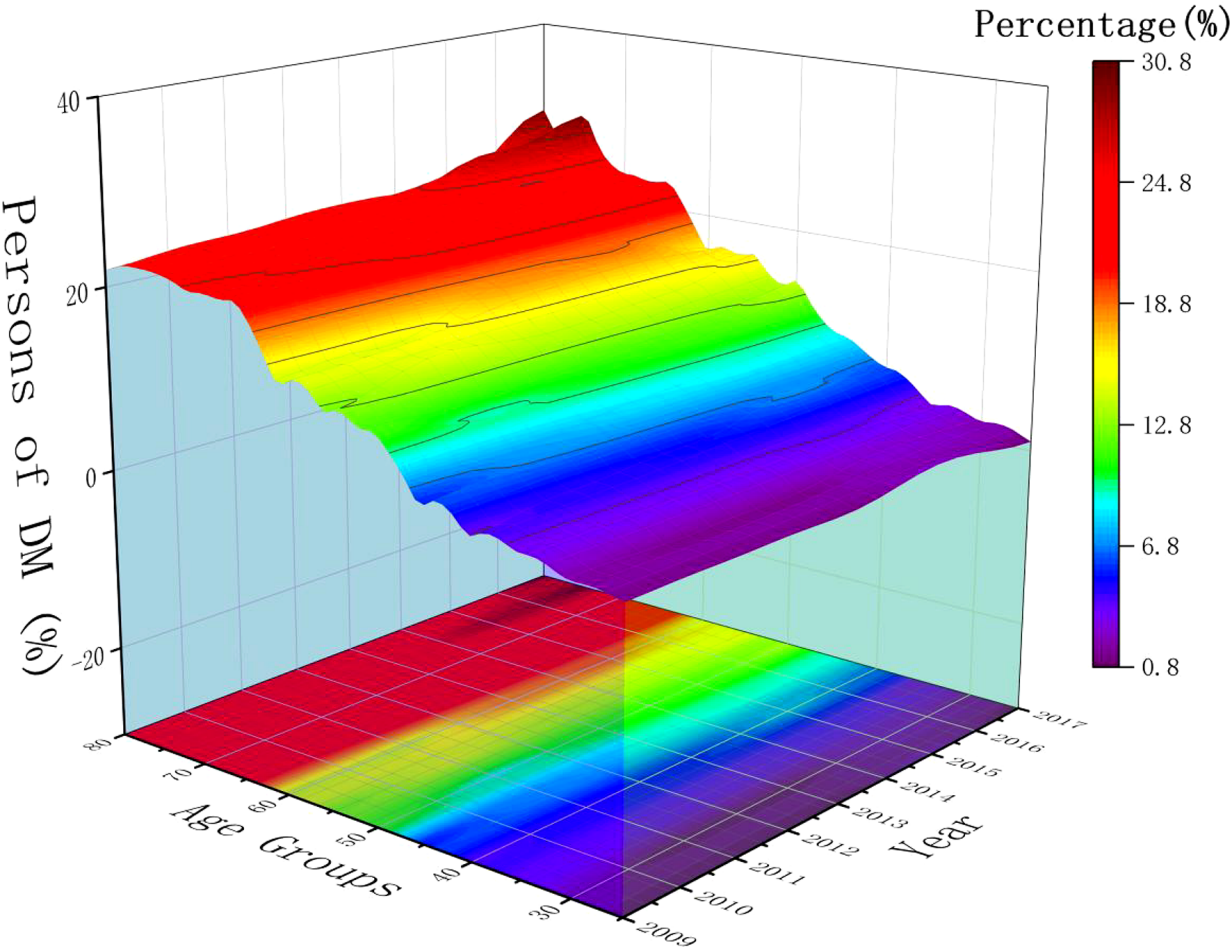
The trend of diabetes incidence from 2009 to 2018 by age groups and year.

The 23 546 NHANES participants with valid body measures and TCHOL value represented 308 million non-institutionalized residents of the United States. Among 26 147 participants with missing data, 30% were white, 24% were black, and 20% were Mexican American. Over the past decade, the prevalence of diabetes has been steadily rising, as is the trend in different races, gender, and age groups (**Figure 2**).

### Demographic characteristics

**Figure 3:**
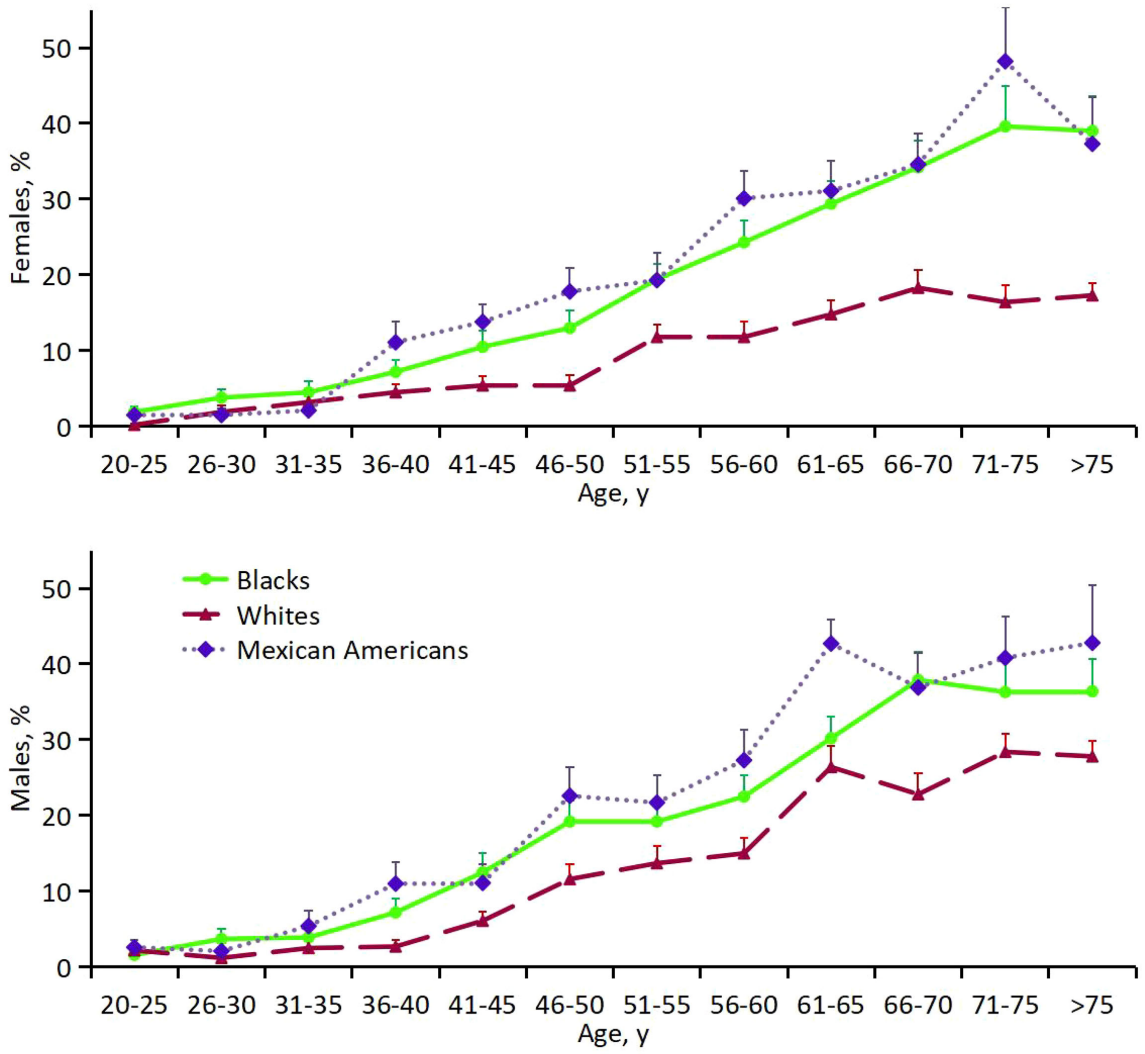
Percentage of females (top) and males (bottom) with DM.

Firstly, the prevalence of diabetes differed by age, sex, and ethnicity (**Figure 3**; **Appendix Table 1**). A total of 3 621 participants had diabetes. The weighted prevalence was 11.6% (95% CI, 11.0% to 12.1%), which represented an estimated 23.1 million persons in the United States. Diabetes was present in 14.6% (CI, 13.6% to 15.6%) of black participants, 10.6% (CI, 9.9% to 11.3%) of white participants, and 13.5% (CI, 11.9% to 15.2%) of Mexican-American participants. Across ethnic groups, white and Mexican-American males were more likely to have diabetes: 12.2% for white males versus 9.1% for white females and 13.7% for Mexican-American males versus 13.4% for Mexican-American females; black men had roughly the same odds of developing diabetes as black women, with only slight differences: 14.5% for black males versus 14.7% for black females. The prevalence of diabetes is increasing with age, males peaked around the 60s, compared to women around the 70s. For every age and sex category, black participants were more likely than white participants to have diabetes; in most age and sex categories, diabetes was more common among Mexican-American participants than among white participants.

Secondly, multivariate logistic regression analysis was done to estimate the prevalence of diabetes adjusted for age, sex, and ethnicity (**Appendix Table 2**). Compared with white participants, black participants and Mexican-American were both more likely (P<0.001) to have diabetes: 14.6% among black participants, 10.6% among white participants, and 13.5% among Mexican-American participants. The prevalence of diabetes was lower in adolescence and was less than 8% from age 20 to 45 years onward. Males were also more likely than females to have diabetes (P<0.001). There was significant interaction between ethnicity and sex (P<0.001) and between ethnicity and age as a continuous variable (P=0.00129).

Thirdly, the prevalence of diabetes with 3 categories fasting plasma glucose (FPG) values was also seen across most age ranges in black participants (**Appendix Table 3**). Most participants with diabetes had fasting plasma glucose (FPG) of more than 125 mg/dL. 67% of black participants, 80% of white participants, and 78% of Mexican-American participants.

### Physical indicators

**Figure 4:**
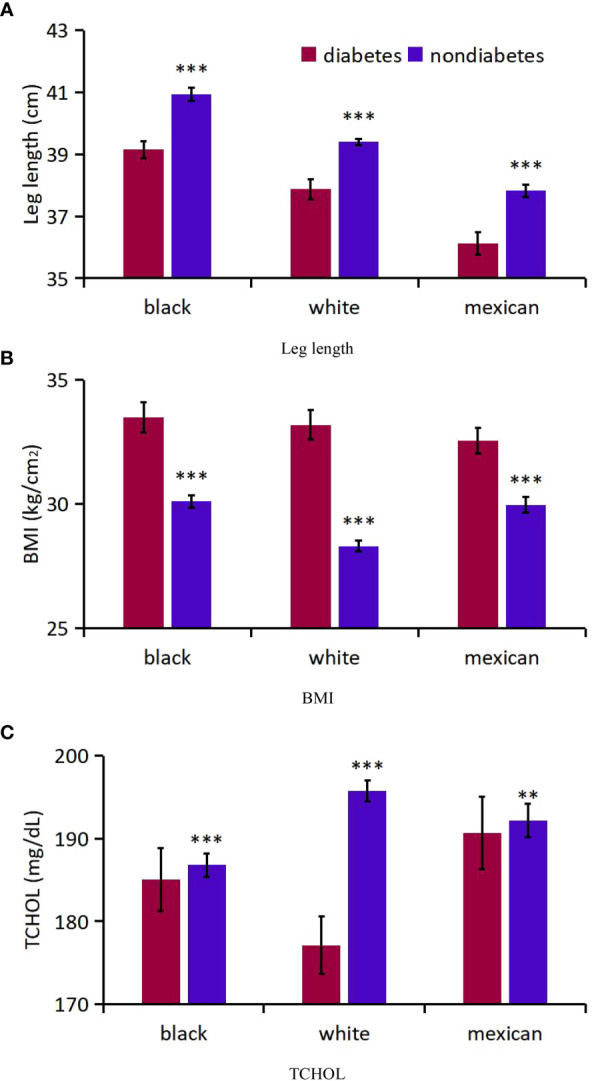
Analysis on the differences of three indicators between diabetic patients and non-diabetic patients of different races. **(A)** Leg length; **(B)** BMI; **(C)** TCHOL. *** P<0.001 vs diabetes; ** P<0.01.

Firstly, mean age-adjusted and sex-adjusted leg length, BMI and TCHOL were compared for diabetics and non-diabetics (**Table 1**, **Figure 4**). The respective numbers of participants who had DM and those who did not have DM were 870 and 3 960 for black, 1 213 and 8 226 for white, and 656 and 2 863 for Mexican-American participants. The overall mean leg length and TCHOL was lower in diabetics than in non-diabetics (1.07 cm, 18.67 mg/dL, respectively), while mean BMI were higher in diabetics than in non-diabetics (4.27 kg/cm^2^). DM had the greatest effect on decline of TCHOL in white participants (23.6 mg/dL), less of an effect in black participants (9.67 mg/dL), and the least effect in Mexican-American participants (8.25 mg/dL).

**Table 1:** Comparative mean leg length, BMI and TCHOL in diabetics and non-diabetics.

Variable	Diabetics	Non diabetics	Mean Difference between diabetics and non-diabetics	P Value
Leg length, cm				
Black	39.2 (38.9-39.4)	40.9 (40.7-41.1)	0.91 (0.67-1.15)	<0.001
White	37.9 (37.5-38.2)	39.4 (39.3-39.5)	1.13 (0.91-1.34)	<0.001
Mexican-American	36.1 (35.8-36.5)	37.8 (37.6-38.0)	0.63 (0.39-0.87)	<0.001
BMI, kg/cm^2^				
Black	33.5 (32.9-34.1)	30.1 (29.8-30.4)	3.49 (2.72-4.25)	<0.001
White	33.2 (32.6-33.8)	28.3 (28.1-28.5)	4.77 (4.41-5.41)	<0.001
Mexican-American	32.5 (32.0-33.1)	30.0 (29.6-30.3)	2.50 (1.86-313)	<0.001
TCHOL, mg/dL				
Black	185.0 (181.3-188.8)	186.8 (185.4-188.2)	9.67 (5.97-13.4)	<0.001
White	177.1 (173.6-180.5)	195.7 (194.4-197.0)	23.6 (20.3-26.9)	<0.001
Mexican-American	190.7 (186.3-195.1)	192.1 (190.1-194.1)	8.25 (2.88-13.6)	0.0037

Secondly, for participants with DM, we compared age-adjusted and sex-adjusted mean leg length, BMI and TCHOL across the major ethnic groups by using linear regression. The numbers of males younger than age 50 years were 1 150, 2 220, and 954among black, white, and Mexican-American participants, respectively, and the numbers of males age 50 years or older were 1 238, 2 490, and 764, respectively. The numbers of females younger than age 50 years were 1 275, 2 248, and 1 0351among black, white, and Mexican-American participants, and the numbers of females age 50 years or older were 1 167, 2 481, and 766, respectively. Relative to white participants, black participants had higher mean leg length (1.28 cm; P<0.001) and higher mean TCHOL (7.96 mg/dL; P=0.0025). Mexican-American participants had lower mean leg length (1.74 cm; P<0.001), lower mean BMI (0.64 kg/m^2^; P=0.11) and apparently higher mean TCHOL (13.61 mg/dL; P<0.001). In addition, mean leg length and TCHOL were higher, and BMI were lower in males than in females (p<0.001) (**Appendix Table 4**). **Appendix Table 5** summarize these 3 variables in the 3 major ethnic groups for sex and for the 12 age groups.

**Figure 5:**
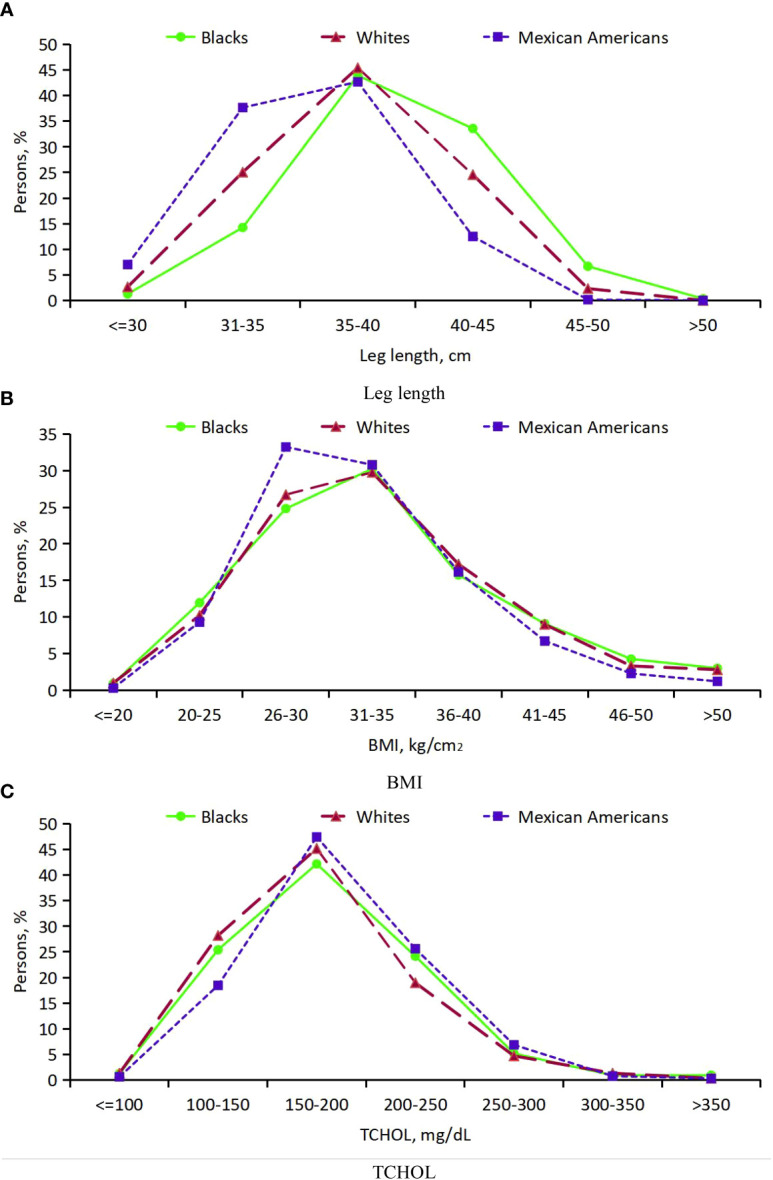
Distribution of leg length, BMI and TCHOL in persons with DM age 20 years or older from 3 ethic groups. **(A)** Leg length; **(B)** BMI; **(C)** TCHOL.

Thirdly, for participants with DM, the distribution of leg length, BMI and TCHOL suggested that, black participants have an advantage in leg length, followed by white participants and Mexican-American participants and there was a downward shift for leg length and a obviously upward shift for BMI among Mexican-American participants, but little difference for TCHOL (**Appendix Table 6**; **Figure 5**).

### Living habits

**Figure 6:**
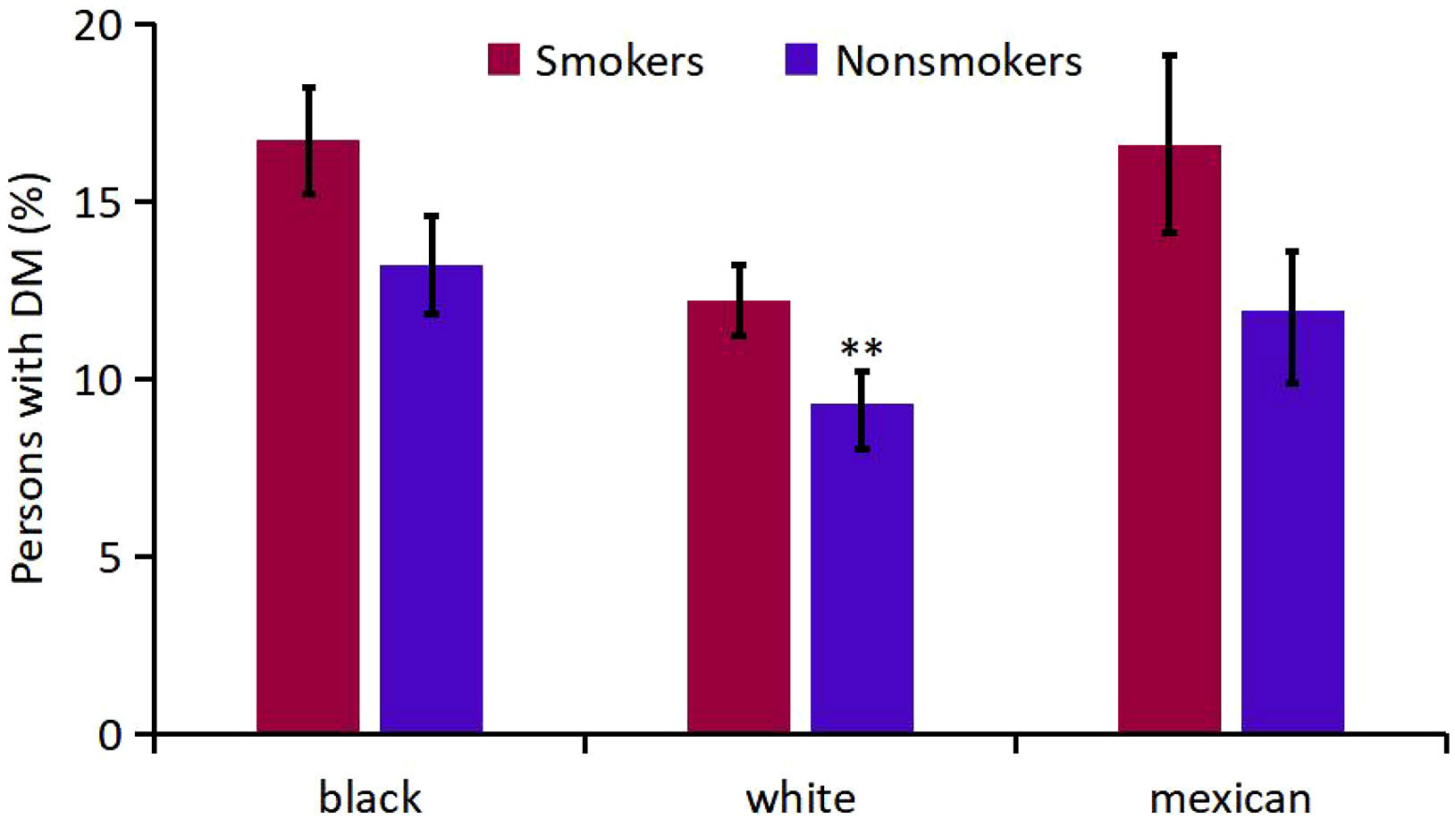
Analysis on the Mean percentage of participants with DM in smokers and nonsmokers of different races. ** P<0.01 vs Smokers

Firstly, mean age-adjusted and sex-adjusted percentage of DM were compared for smokers and nonsmokers (**Appendix Table 7**, **Figure 6**). The respective numbers of participants who had smoked and those who did not smoke were 2 084 and 2 746 for black, 4 880 and 4 559 for white, and 1 272 and 2247 for Mexican-American participants. There was no significant difference in the overall mean percentage of DM between smokers and non-smokers (P=0.13). Meanwhile, smoking had great effect on percent increment of DM in white participants (0.2%), and have little effect on black and Mexican-American participants. In a separate logistic regression analysis that was adjusted for age group, sex, and ethnicity, smokers had a statistically significant lower percentage of DM (CI, 0.07 to 0.28; P=0.002) relative to nonsmokers.

**Figure 7:**
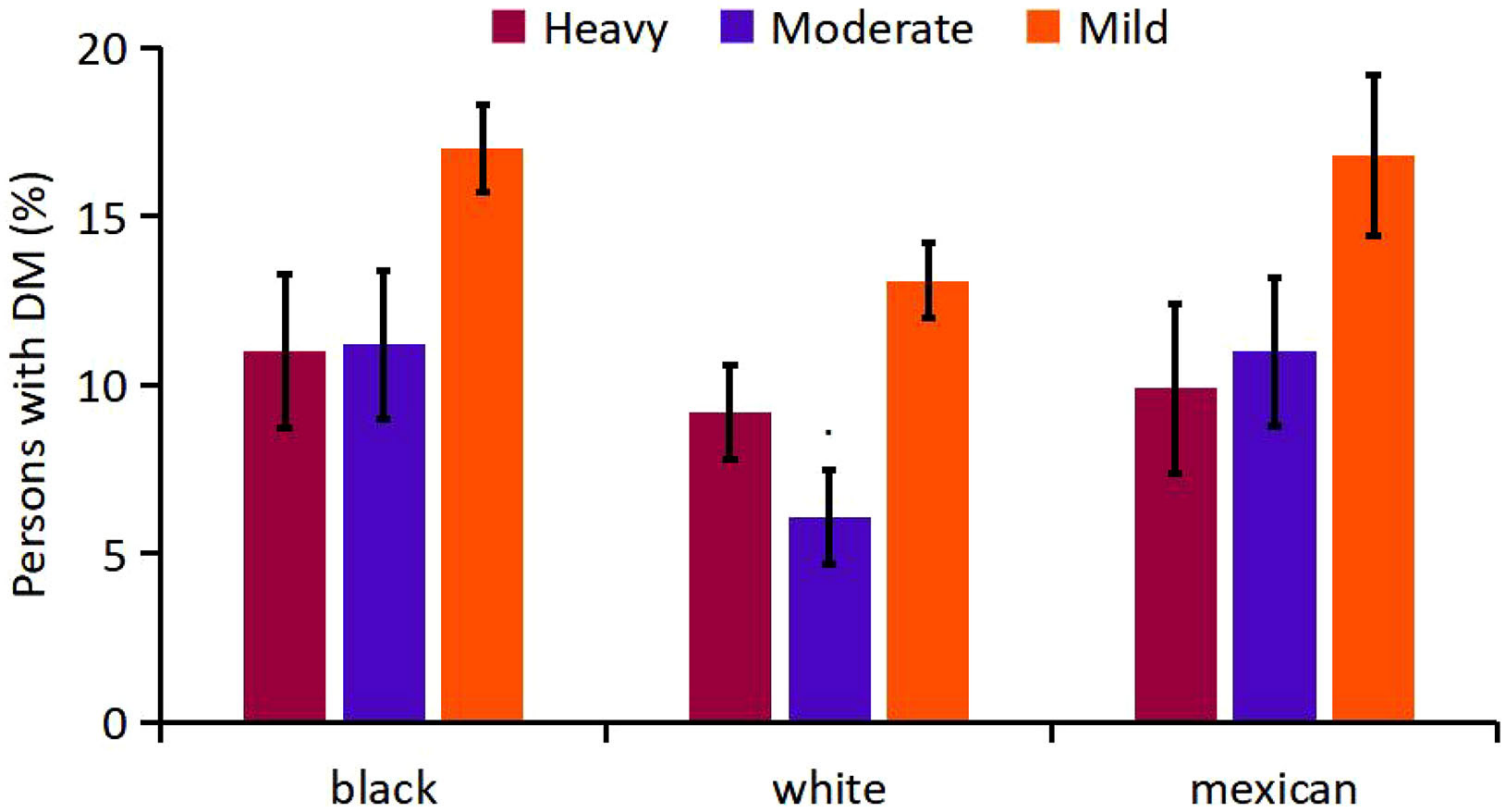
Analysis on the Mean percentage of participants with DM in alcohol-drinking groups of different races.

Secondly, mean age-adjusted and sex-adjusted percentage of DM were compared for alcohol-drinking groups (**Appendix Table 8**, **Figure 7**). The respective numbers of participants who were heavy, moderate, mild alcohol-drinkers were 8 51, 9 88 and 2 991 for black, 1 709, 2 044 and 5 686 for white, and 709, 880 and 1 930 for Mexican-American participants. Interestingly, heavy and moderate alcohol-drinkers had lower mean prevalence of DM than mild alcohol-drinkers. The association of moderate drinking with diabetes was significantly different from mild drinking (P=0.004), otherwise, the differences among the alcohol-drinking groups were not obvious.

## Discussion

For demographic characteristics, reports have confirmed that the biological factors affecting the pathophysiology of diabetes differ from race or nationality, and may be influenced by social factors, such as different cultural backgrounds (17–24). Data from the Centers for Disease Control and Prevention shows that the probability of some racial and ethnic groups being diagnosed with diabetes in the USA is much higher than that of whites, which was reviewed in Lancet (25). At the moment of the COVID-19 pandemic, the risk of diabetes among the black population has further increased, and is inextricably linked with income, education, occupation, housing, food security, social support and other factors (25). Our analysis overcame the problem of insufficient data in the past to extend population-based observations that the prevalence of diabetes differ by sex and age. Males are slightly more likely to have diabetes than female counterparts, which was also confirmed by a recent study on the mechanism of gender difference inducing diabetes (26). In addition, prevalence of diabetes were lower in younger persons. These sex- and age-related differences in percentage of participants with diabetes were consistently seen in all 3 major ethnic groups. Among the basic 3 factors, ethnicity is more closely related with diabetes and is one of the factors that increase its incidence. The current analysis shows that DM is most common among male black elderly, and certain reports have described similar findings (26, 27). This population-based report suggests broad genetic influences. The findings in the current analysis were consistent and robust for the observed differences in age, sex, and ethnicity (24, 28–31).

In addition, most participants with DM had fasting plasma glucose (FPG) of more than 125 mg/dL. Due to the different criteria for diabetes (7, 14, 32–35), the standard we adopted (14) is consistent with AACE/ACE standard (32), which is widely recognized. Although it is tempting to suggest that the NHANES database should collect data on diabetes with reference to a given standard, such a recommendation may be premature. The detailed clinical data of NHANES have its own considerations, with considerable independence and objectivity, therefore, the research reference criteria of different researchers may vary, and a reasonable and valid judgment criterion needs to be further confirmed.

For physical indicators, our study shows that people with diabetes have shorter legs than those without diabetes, which confirms the findings from a cross-sectional analysis of data about simple-measured leg muscle strength and the prevalence of diabetes among Japanese males (36). Some studies have shown that stretching the leg can reduce the incidence of diabetes (37–39). BMI is a comprehensive embodiment of weight and height, which has certain reference value for studying the influence of obesity and overweight on diabetes. Our finding of a higher BMI being associated with diabetes prevalence is consistent with the known effectiveness of weight control in treating diabetes (30, 31). Interestingly, in the study TCHOL tends to be lower in diabetic patients, and little research on this area (40). In addition, for participants with DM, the distribution of leg length, BMI and TCHOL varies among ethnic groups, which also illustrates the association of race with diabetes.

With regard to living habits, smoking had a modest association with diabetes prevalence. This finding was also seen in a prospective study published (41). Smoking may increase the risk of diabetes through a variety of mechanisms, with tobacco-induced insulin resistance (IR) and hyperinsulinism (HIS) as the main mechanisms. Currently the mechanism of smoking causing and aggravating IR is not completely concluded. It is quite consistent that chronic smoking may lead to lipid metabolism disorders, increased abdominal obesity and vascular endothelial dysfunction (41–46). Smoking statistically had a large effect on the increased percentage of DM among white participants and had minimal effect on black and Mexican-American participants. To our knowledge, this may be the first report of such an observation, and its significance is unknown.

As for alcohol, interestingly, heavy and moderate drinkers had lower mean prevalence of DM than mild drinkers and the association of moderate drinking with DM was significantly different from mild drinking. A large meta-analysis illustrated a similar effect that reductions in risk of DM among moderate alcohol drinkers may be confined to women and non-Asian populations (47).

There are some limitations in our study. Firstly, the study focuses mainly on the prevalence of diabetes and did not explore associated complications. Secondly, the data on fasting plasma glucose is relatively limited, resulting in a large reduction in the sample size in the classification of statistics. Thus, the results of the analysis based on these data may be exaggerated. However, the prevalence of diabetes among ethnic groups is probably not affected, because participants with missing data were evenly distributed among all 3 groups. Third, the observed relationships were limited to adults of 3 races in the US and not include other races, particularly Asian-Americans, which may have limited the extrapolation of the results. Given these limitations, a well-designed prospective cohort trial is needed to validate our results.

## Conclusions

It can be concluded that DM is more common in the general population than might be clinically recognized, and the prevalence of DM was associated to varying degrees with many indicators of demographic characteristics (race, gender, age), physical indicators (leg length, BMI, TCHOL), and living habits (smoking, alcohol-drinking). When deciding whether a thorough diagnostic assessment of diabetes is needed, not only the clinical symptoms should be considered, but also these indicators. They should be linked with medical resource allocation and scientific treatment methods to comprehensively implement the treatment of DM.

## Data availability statement

Publicly available datasets were analyzed in this study. This data can be found here: https://www.cdc.gov/nchs/nhanes/index.htm.

## Author contributions

LF was involved in all parts of the study, design, acquisition of data, analysis, interpretation, drafting of paper, and final approval. HS and YT participated in data analysis and helped to revise the manuscript. QZ coordinated and supervised data collection, and critically reviewed the manuscript for important intellectual content. All authors approved the final version as submitted, and agree to be accountable for all aspects of the work.
