# Supplementary material for: Prevalence of diabetes in the USA from the perspective of demographic characteristics, physical indicators and living habits based on NHANES 2009-2018
Source: Front Endocrinol (Lausanne). 2023 Mar 7;14:1088882. doi: 10.3389/fendo.2023.1088882 (PMC10028205; doi:10.3389/fendo.2023.1088882)
Supplement: Supplementary file 1 [file DataSheet_1.docx]

**Appendix Table 1. Percentage of Male and Female Participants with diabetes**

| Sex and Age Group | Participants, n(%) [95% CI] | | |
| --- | --- | --- | --- |
|  | Black | White | Mexican-American |
| **Males** |  |  |  |
| 20-25 y | 4( 1.6) [ 0.1- 3.2] | 11( 2.2) [ 0.6- 3.8] | 6( 2.6) [ 0.6- 4.6] |
| 26-30 y | 6( 3.7) [ 1.0- 6.4] | 6( 1.2) [ 0.5- 2.0] | 4( 2.1) [ 0- 4.1] |
| 31-35 y | 8( 3.9) [ 1.3- 6.6] | 12( 2.5) [ 0.7- 4.3] | 8( 5.4) [ 1.4- 9.5] |
| 36-40 y | 12( 7.2) [ 3.7-10.7] | 13( 2.7) [ 0.8- 4.5] | 19(11.0) [ 6.1-16.0] |
| 41-45 y | 24(12.5) [ 7.7-17.4] | 29( 6.1) [ 3.7- 8.5] | 16(11.1) [ 6.1-16.0] |
| 46-50 y | 40(19.2) [13.7-24.7] | 46(11.6) [ 7.8-15.5] | 33(22.6) [15.0-30.1] |
| 51-55 y | 43(19.2) [14.2-24.2] | 62(13.7) [ 9.1-18.2] | 37(21.7) [14.7-28.7] |
| 56-60 y | 53(22.5) [17.0-28.1] | 56(15.0) [10.9-19.0] | 45(27.3) [19.4-35.2] |
| 61-65 y | 87(30.2) [24.4-36.0] | 93(26.4) [21.1-31.8] | 78(42.7) [36.4-49.0] |
| 66-70 y | 75(37.9) [30.7-45.2] | 79(22.8) [17.2-28.3] | 37(36.9) [27.9-46.0] |
| 71-75 y | 39(36.3) [27.3-45.3] | 109(28.4) [23.7-33.0] | 32(40.8) [29.9-51.6] |
| >75 y | 55(36.4) [27.9-44.9] | 181(27.8) [23.8-31.8] | 25(42.8) [27.9-57.7] |
| **Females** |  |  |  |
| 20-25 y | 5( 2.0) [ 0.5- 3.3] | 1(0.2) [-0.2-0.6] | 3( 1.5) [-0.2-3.2] |
| 26-30 y | 8( 3.8) [ 1.6- 5.9] | 7(1.9) [ 0.4-3.4] | 4( 1.5) [-0.3-3.3] |
| 31-35 y | 9( 4.5) [ 1.5- 7.5] | 10(3.2) [ 0.6-5.7] | 4( 2.1) [ 0- 4.3] |
| 36-40 y | 15( 7.2) [ 4.0-10.4] | 23(4.5) [ 2.4-6.7] | 21(11.1) [ 5.7-16.5] |
| 41-45 y | 26(10.5) [ 6.2-14.8] | 28(5.4) [ 3.0-7.8] | 28(13.8) [ 9.3-18.4] |
| 46-50 y | 28(13.0) [ 8.4-17.5] | 27(5.4) [ 2.6-8.2] | 30(17.8) [11.7-24.0] |
| 51-55 y | 47(19.4) [ 1.5-23.3] | 52(11.8) [ 8.7-14.9] | 29(19.3) [12.2-26.4] |
| 56-60 y | 60(24.3) [18.6-30.0] | 50(11.8) [ 7.7-15.9] | 45(30.1) [23.1-37.0] |
| 61-65 y | 81(29.4) [23.4-35.3] | 66(14.8) [11.2-18.3] | 54(31.1) [23.4-38.9] |
| 66-70 y | 55(34.2) [27.4-41.1] | 72(18.3) [13.7-22.9] | 46(34.6) [26.8-42.4] |
| 71-75 y | 38(39.6) [29.2-50.1] | 60(16.4) [12.2-20.7] | 28(48.2) [34.1-62.4] |
| >75 y | 52(39.0) [29.9-48.1] | 120(17.2) [14.1-20.4] | 24(37.3) [25.1-49.5] |

**Appendix Table 2. Prevalence of diabetes**

| Variable | Prevalence, %(95% CI) |
| --- | --- |
| **Ethnicity** |  |
| White | 10.6( 9.9-11.3) |
| Black | 14.6(13.6-15.6) |
| Mexican American | 13.5(11.9-15.2) |
| **Sex** |  |
| Female | 10.4( 9.7-11.1) |
| Male | 12.7(11.9-13.5) |
| **Age** |  |
| 20-25 y | 1.5( 0.9- 2.1) |
| 26-30 y | 1.8( 1.3- 2.4) |
| 31-35 y | 3.3( 2.5- 4.1) |
| 36-40 y | 5.2( 4.1- 6.3) |
| 41-45 y | 7.7( 6.4- 8.9) |
| 46-50 y | 11.4( 9.5-13.4) |
| 51-55 y | 14.5(12.3-16.7) |
| 56-60 y | 16.0(13.6-18.5) |
| 61-65 y | 23.4(20.9-25.8) |
| 66-70 y | 23.3(20.3-26.4) |
| 71-75 y | 25.7(23.0-28.5) |
| >75 y | 24.2(22.1-26.3) |

**Appendix Table 3. Overall Prevalence of Black Participants with diabetes of different FPG groups**

| Age Group | Participants with FPG<100 mg/dL | Participants with FPG of 100-125 mg/dL | Participants with FPG >125 mg/dL |
| --- | --- | --- | --- |
| 20-25 y | 190(81.6) [77.1-86.2] | 42(17.2) [12.7-21.8] | 2( 1.1) [ 0- 2.7] |
| 26-30 y | 143(75.4) [68.9-82.0] | 40(20.6) [14.5-26.7] | 8( 4.0) [ 1.7- 6.4] |
| 31-35 y | 115(63.2) [55.0-71.5] | 59(32.2) [25.1-39.3] | 9( 4.6) [ 1.5- 7.6] |
| 36-40 y | 101(63.1) [55.5-70.6] | 48(29.1) [22.5-35.6] | 13( 7.8) [ 3.5-12.2] |
| 41-45 y | 106(54.1) [46.0-62.2] | 67(36.5) [28.3-44.6] | 20( 9.5) [ 5.1-13.8] |
| 46-50 y | 82(45.4) [37.7-53.2] | 76(39.5) [32.3-46.8] | 30(15.0) [ 9.6-20.5] |
| 51-55 y | 107(46.5) [39.3-53.7] | 99(43.5) [36.5-50.5] | 23(10.0) [ 6.4-13.7] |
| 56-60 y | 96(43.1) [35.7-50.6] | 96(39.2) [32.4-45.9] | 42(17.7) [12.8-22.6] |
| 61-65 y | 89(38.0) [31.5-44.4] | 105(37.5) [31.3-43.7] | 66(24.5) [18.4-30.7] |
| 66-70 y | 45(26.3) [18.7-33.9] | 83(50.5) [41.1-59.8] | 42(23.2) [16.7-29.8] |
| 71-75 y | 28(33.2) [24.0-42.4] | 36(36.4) [24.8-48.0] | 26(30.4) [19.9-40.9] |
| >75 y | 50(35.6) [28.5-42.7] | 61(42.1) [33.1-51.1] | 34(22.2) [16.1-28.4] |

**Appendix Table 4. Mean body measures and TCHOL values, by Age, Sex, and Ethnic Group**

| Sex and Age Group | Participants,n | Leg length, cm | BMI, kg/cm^2^ | TCHOL, mg/dL |
| --- | --- | --- | --- | --- |
| **Black** |  |  |  |  |
| All | 870 | 39.2≠(38.9-39.4) | 33.5\|\|(32.9-34.1) | 185.0¶(181.3-188.8) |
| Males <50 y | 88 | 42.8\|\|(42.1-43.4) | 34.1\|\|(32.2-35.9) | 192.9\|\|(181.1-204.6) |
| Males ≥50 y | 358 | 41.2≠(40.9-41.6) | 31.7\|\|(31.0-32.4) | 173.5¶(169.0-178.0) |
| Females <50 y | 85 | 38.2¶(37.6-38.9) | 35.7\|\|(33.9-37.5) | 193.4\|\|(183.7-203.1) |
| Females ≥50 y | 339 | 36.6≠(36.2-36.9) | 34.0\|\|(33.1-34.8) | 188.6\|\|(183.0-194.2) |
| **White** |  |  |  |  |
| All | 1213 | 37.9(37.5-38.2) | 33.2(32.6-33.8) | 177.1(173.6-180.5) |
| Males <50 y | 106 | 41.9(41.1-42.8) | 33.8(31.8-35.7) | 185.4(176.9-193.9) |
| Males ≥50 y | 591 | 39.8(39.5-40.1) | 32.1(31.3-32.8) | 165.2(160.7-169.8) |
| Females <50 y | 90 | 36.6(35.8-37.4) | 36.6(34.7-38.6) | 187.9(175.3-200.6) |
| Females ≥50 y | 426 | 34.5(34.2-34.8) | 33.7(32.9-34.5) | 187.8(183.0-192.7) |
| **Mexican Americans** |  |  |  |  |
| All | 656 | 36.1≠(35.8-36.5) | 32.5\|\|(32.0-33.1) | 190.7≠(186.3-195.1) |
| Males <50 y | 75 | 39.4≠(38.9-39.9) | 31.7\|\|(30.6-32.9) | 206.5¶(197.9-215.1) |
| Males ≥50 y | 265 | 38.1≠(37.6-38.6) | 30.8§(30.3-31.4) | 177.5¶(170.9-184.2) |
| Females <50 y | 83 | 34.2≠(33.6-34.7) | 35.0\|\|(33.3-36.8) | 195.1\|\|(183.0-207.1) |
| Females ≥50 y | 233 | 32.7≠(32.4-33.1) | 33.4\|\|(32.4-34.5) | 190.5\|\|(184.6-196.3) |
| * Means (95% CI) are given from a weighted analysis adjusted for age and sex unless otherwise indicated.  ≠ P < 0.001 compared with white participants.  ¶ P ≤ 0.001 to < 0.01 compared with white participants.  § P < 0.01 to < 0.05 compared with white participants.  \|\| P > 0.05 compared with white participants. | | | | |

**Appendix Table 5. Leg length, BMI and TCHOL values in 3 Major Ethnic Groups, by Age**

| Sex and Age Group | Participants, n(%) [95% CI] | | |
| --- | --- | --- | --- |
|  | Black | White | Mexican-American |
| **Leg length, cm** |  |  |  |
| 20-25 y | 40.8(39.0-42.6) | 43.1(42.3-43.9) | 38.7(38.1-39.2) |
| 26-30 y | 42.0(40.1-44.0) | 41.1(38.7-43.4) | 38.6(35.9-41.2) |
| 31-35 y | 40.2(38.8-41.7) | 39.0(37.5-40.4) | 39.3(37.2-41.3) |
| 36-40 y | 40.3(39.0-41.6) | 38.1(35.4-40.7) | 37.1(35.9-38.2) |
| 41-45 y | 40.1(38.9-41.4) | 38.1(37.0-39.1) | 36.5(35.2-37.8) |
| 46-50 y | 40.0(39.1-41.0) | 40.4(39.1-41.6) | 36.2(35.1-37.3) |
| 51-55 y | 39.4(38.5-40.4) | 38.0(37.0-38.9) | 36.1(35.1-37.1) |
| 56-60 y | 39.2(38.4-40.0) | 38.2(37.3-39.1) | 35.8(34.6-37.0) |
| 61-65 y | 39.0(38.3-39.6) | 37.5(36.8-38.1) | 35.7(35.0-36.3) |
| 66-70 y | 38.3(37.6-39.1) | 37.2(36.5-37.8) | 34.7(33.8-35.6) |
| 71-75 y | 37.3(36.1-38.5) | 37.1(36.5-37.7) | 35.2(34.1-36.4) |
| >75 y | 37.8(37.1-38.5) | 37.0(36.6-37.5) | 34.6(33.6-35.7) |
| **BMI, kg/cm2** |  |  |  |
| 20-25 y | 33.0(27.8-38.2) | 37.3(28.6-46) | 29.8(25.1-34.5) |
| 26-30 y | 36.4(30.1-42.7) | 32.9(25.2-40.6) | 35.2(31.6-38.9) |
| 31-35 y | 36.8(31.4-42.2) | 32.8(28.5-37.1) | 31.8(28.3-35.3) |
| 36-40 y | 34.1(31.4-36.9) | 35.7(29.9-41.4) | 33.5(31.1-36) |
| 41-45 y | 35.8(34.0-37.5) | 36.8(34.6-39.1) | 35(33-36.9) |
| 46-50 y | 34.4(32.5-36.3) | 34.3(32.7-35.9) | 32.1(30.7-33.6) |
| 51-55 y | 34.5(33-36.1) | 34.5(32.6-36.4) | 33.5(31.8-35.1) |
| 56-60 y | 35.7(34.4-37) | 34.5(33-36.1) | 33.6(32.1-35.1) |
| 61-65 y | 32.4(31.5-33.3) | 33.6(32.6-34.6) | 31.1(30.2-31.9) |
| 66-70 y | 32(31-33) | 32.8(31.6-34) | 32.3(30.7-33.9) |
| 71-75 y | 31.2(29.6-32.9) | 31.8(30.9-32.7) | 30.3(28.7-31.8) |
| >75 y | 29.4(28.1-30.8) | 29.7(29-30.3) | 30(29-31) |
| **TCHOL, mg/dL** |  |  |  |
| 20-25 y | 175.2(136.1-214.2) | 175.9(145.7-206.1) | 183(167-199) |
| 26-30 y | 218(194.2-241.7) | 176(154.4-197.6) | 223.1(198.4-247.7) |
| 31-35 y | 216.8(184.5-249.1) | 172.2(146.5-197.9) | 216.4(195.1-237.6) |
| 36-40 y | 188.7(166.6-210.7) | 195.6(175.5-215.8) | 207.3(188.4-226.2) |
| 41-45 y | 188(174.6-201.3) | 193.1(177.9-208.3) | 187.7(176.8-198.5) |
| 46-50 y | 187.9(178.8-197.1) | 184.5(174-195.1) | 205.5(192.1-218.8) |
| 51-55 y | 182.5(172.3-192.7) | 181.8(172.1-191.4) | 185.5(175.5-195.5) |
| 56-60 y | 187.7(177.4-198) | 187.7(177.2-198.3) | 187.1(176.3-197.9) |
| 61-65 y | 185.5(178.7-192.4) | 178.8(172.2-185.4) | 181.6(174-189.1) |
| 66-70 y | 176.4(167.2-185.6) | 170.6(160.2-181.1) | 175.1(166.9-183.4) |
| 71-75 y | 179.4(161.6-197.1) | 172.1(163.4-180.8) | 186.6(172.1-201) |
| >75 y | 175.6(166.2-185.1) | 161.6(156.9-166.2) | 168.4(156.6-180.1) |
| * Unadjusted means (95% CI) are given unless otherwise indicated. Age groups are based on strata that are commonly used in highly stratified National Health and Nutrition Examination Survey analyses. No statistical comparisons were done for this table. | | | |

**Appendix Table 6. Number and Proportion of Participants, by Distribution of TCHOL and BMI.**

(a) Leg length

| Ethnic Group |  |  | Leg length, cm | | |  |
| --- | --- | --- | --- | --- | --- | --- |
|  | <=30 | 30-35 | 35-40 | 40-45 | 45-50 | >50 |
| Black, n(%) | 1.26(11) | 14.25(124) | 43.91(382) | 33.56(292) | 6.67(58) | 0.34(3) |
| White, n(%) | 2.64(32) | 25.06(304) | 45.42(551) | 24.57(298) | 2.31(28) | 0(0) |
| Mexican-American, n(%) | 7.01(46) | 37.65(247) | 42.68(280) | 12.5(82) | 0.15(1) | 0(0) |

(b) BMI

| Ethnic Group |  |  | BMI, kg/cm^2^ | | |  |  |  |
| --- | --- | --- | --- | --- | --- | --- | --- | --- |
|  | <=20 | 20-25 | 26-30 | 31-35 | 36-40 | 41-45 | 46-50 | >50 |
| Black, n(%) | 0.92(8) | 11.95(104) | 24.83(216) | 30.23(263) | 15.75(137) | 9.08(79) | 4.25(37) | 2.99(26) |
| White, n(%) | 0.99(12) | 10.22(124) | 26.71(324) | 29.76(361) | 17.23(209) | 8.99(109) | 3.3(40) | 2.8(34) |
| Mexican-American, n(%) | 0.3(2) | 9.3(61) | 33.23(218) | 30.79(202) | 16.16(106) | 6.71(44) | 2.29(15) | 1.22(8) |

(c) TCHOL

| Ethnic Group | TCHOL, mg/dL | | | | | | |
| --- | --- | --- | --- | --- | --- | --- | --- |
|  | <=100 | 100-150 | 150-200 | 200-250 | 250-300 | 300-350 | >350 |
| Black, n(%) | 1.26(11) | 25.4(221) | 42.18(367) | 24.14(210) | 5.17(45) | 0.92(8) | 0.92(8) |
| White, n(%) | 1.32(16) | 28.19(342) | 45.18(548) | 18.96(230) | 4.7(57) | 1.32(16) | 0.33(4) |
| Mexican-American, n(%) | 0.61(4) | 18.45(121) | 47.41(311) | 25.61(168) | 6.86(45) | 0.76(5) | 0.3(2) |

**Appendix Table 7. Comparative Mean percentage of participants with DM in smokers and nonsmokers**

| **Variable** | **Smokers** | **Nonsmokers** | **Mean Difference between diabetics and non diabetics** | **P Value** |
| --- | --- | --- | --- | --- |
| Black | 16.7(15.2-18.2) | 13.2(11.9-14.6) | 0.08(-010-0.25) | 0.38 |
| White | 12.2(11.2-13.2) | 9.3( 8.3-10.2) | 0.20( 0.06-0.35) | 0.0089 |
| Mexican-American | 16.6(14.1-19.1) | 11.9(10.2-13.6) | 0.14(-0.07-0.36) | 0.19 |

**Appendix Table 8. Comparative Mean percentage of participants with DM in alcohol-drinking groups**

| **Variable** | **Heavy** | **Moderate** | **Mild** |
| --- | --- | --- | --- |
| Black | 11 (8.6-13.3) | 11.2\|\|(9 -13.5) | 17 \|\|(15.7-18.4) |
| White | 9.2(7.8-10.6) | 6.1 \|\|(4.7 - 7.5) | 13.1\|\|(12 -14.3) |
| Mexican-American | 9.9(7.4-12.4) | 11 \|\|(8.8-13.2) | 16.8\|\|(14.5-19.2) |
| ≠ P < 0.001 compared with heavy alcohol drinkers.  ¶ P >= 0.001 to < 0.01 compared with heavy alcohol drinkers.  § P > =0.01 to < 0.05 compared with heavy alcohol drinkers.  \|\| P >= 0.05 to < 0.1 compared with heavy alcohol drinkers. | | | |
